# Video corroboration of player incurred impacts using trunk worn sensors among national ice-hockey team members

**DOI:** 10.1371/journal.pone.0218235

**Published:** 2019-06-24

**Authors:** Aaron Pilotti-Riley, Davor Stojanov, Muhammad Sohaib Arif, Stephen J. McGregor

**Affiliations:** Sport Performance Technologies Laboratory, School of Health Promotion & Human Performance, Collage of Health & Human Services, Eastern Michigan University, Ypsilanti, Michigan, United States of America; Instituto Politecnico de Viana do Castelo, PORTUGAL

## Abstract

**Purpose:**

Video corroboration of player incurred impacts (PII) using trunk-worn wearable sensors (WS) among national ice-hockey team members.

**Methods:**

23 members of the U.S. National (NTDP) U18 team consented to procedures approved by EMU Human Subjects Committee. Bioharness-3 (Zephyr, MD) WS recorded occurrences of PII during games and impacts were generated using Impact Processor (Zephyr, MD). Eight players with the top activity levels each game determined by WS, were observed using video and synchronized with game video collected by NTDP staff. Impacts identified by WS of 6–7.9 g (Z3), 8–9.9 g (Z4) and 10+ g (Z5) were used to corroborate PII. Magnitude and duration of each identified impact were compared by category using MANOVA with Tukey *post hoc* (α = 0.05; SPSS 22.0, IBM, NY).

**Results:**

Of 419 on-ice impacts, 358 were confirmed true PII (85.5%), 60 as other non-PII (14.3%) and 1 false positive (0.2%). For 358 PII, 17 (4.1%) were 1) Board contact/no check, 74 (17.7%), 2) Board contact/check, 202 (48.2%), 3) Open ice check, 65 (15.5%), 4) Player fall. Of 60 Non-PII, 19 (4.5%) as 5) other form of player to player event, 16 (3.8%) as 6) Hard Stop, 19 (4.5%) as 7) Slapshots and 6 (1.4%) as 8) other identifiable player events. 160 of the 200 Z3 events were PII (80%), 103 of 110 Z4 events (93.6%) and 95 of 109 Z5 events were PII (87.2%). The magnitude of impacts was not different by category, but the duration of category 6 (Hard stop; .058 s) was lower than categories 2, 4 and 7 (.112, .112, .133 s, respectively, p < .05).

**Conclusion:**

These data show that using some limited criteria (e.g. impact magnitude and duration), PII can be identified with relatively high accuracy in ice hockey using trunk-worn wearable sensors.

## Introduction

Youth sport participation has declined in recent years. There may be several likely contributors to this drop in participation, but, particularly in contact sports, there is increasing concern for athlete safety [1]. As an example, youth tackle football was met with a 29% decline in participation of 6–12 year old’s between 2008 and 2013 [2]. In contrast though, another contact sport, ice-hockey, is the only major sport that has not experienced a decline in youth participation in the eight years up to 2015 [3]. Despite the healthy participation rates in youth ice-hockey relative to other sports, growth is slow and there is still debate about the inclusion of checking in the sport and at what age it is appropriate. To this point, in 2011, USA Hockey raised the minimum age that body checking is permitted from 11–12 years (Pee Wee) to 13–14 years (Bantam level) [4]. To examine the issue of player safety and checking in youth ice-hockey, numerous epidemiological studies have been performed, but the results have been equivocal [5, 6]. Some studies reported that decreasing age of exposure to checking increased the odds of injury [7], while other studies found no significant change to injury rates with an increase to the minimum checking age [8]. With the equivocal nature of epidemiological data, there is a clear need for more quantitative data with regard to actual impacts incurred by players participating in ice-hockey.

Previous data does exist with regard to impacts incurred in ice hockey, but it is generally specific to head impacts [9, 10] as concern about concussions has primarily driven research in this area. Head impacts and traumatic brain injuries are important concerns for athlete safety in all contact sports and some non-contact sports [11–16]. However, other injuries might result from whole body impacts, because it has been argued that the majority of injuries occurring in contact sports actually result from unintentional contact [17]. Some of these impacts might be a result of body checking, while others might be non-checking related impacts such as players making contact with the boards or simply falling on the ice;. Collectively, we term these events player incurred impacts (PII). Each of these impacts could lead to player injuries unrelated to head impacts of concussions. The literature related to the frequency and severity of checking related injuries, indicates that checking can be responsible for 45–86% of injuries for youth playing ice hockey. These injuries result in many weeks of recovery time for concussions or soft tissue injury’s [18]. Finally, impacts in general may influence participation rates in the sport. Therefore, it is of interest to gather quantitative information with regard to the magnitude and frequency of impacts incurred by players during ice-hockey participation.

Whole body impact quantification in sports other than ice-hockey has been performed utilizing wearable sensor accelerometer technology. These devices are especially useful for acquiring data in team sports as they are designed to collect “ecologically valid data” and to be used while the athletes participate in actual competitive game scenarios. Such sensors were used by Gastin et al., to detect tackles and impact events in elite Australian football [19], and by Corties et al., to verify the frequency/magnitude of head impacts during lacrosse games [20]. While wearable sensor technology has been utilized in research for other contact sports (Australian Rules Football, lacrosse) it has mainly been used for sports were running was the primary method of locomotion[19–24]. Less data is available with regard to the use of wearable sensors in the sport of ice hockey. This may potentially be due to the dynamic characteristics of ice hockey in which the primary method of locomotion is skating, or the unique on-ice conditions as these factors can make data analysis challenging [25]. As stated by Upjohn et al., “Unlike dry land walking or running, skating locomotion involves substantial movements in both sagittal and frontal planes. Despite the obvious medial–lateral sinusoidal body migration during skating, limited quantitative measures in this plane have been obtained” [26]. As such there is currently no evidence regarding the objective quantification of impacts incurred in ice-hockey using wearable sensors.

Aside from the general paucity of data generated by wearable sensors in ice-hockey, there is currently no whole-body impact data with regard to ice-hockey. The first step in quantifying impacts in ice-hockey is validating the information provided by wearable sensors used to detect impacts. The Zephyr Bio Harness (Medtronic, MD) brand of wearable sensor provides an impact identification algorithm. This algorithm has been developed for use in other sports, but this system has not been validated in ice-hockey. Validating wearable sensor technology in ice hockey could greatly assist in monitoring the magnitude and frequency of impacts the players are experiencing, thereby assisting in evaluating injury risk, muscle damage, recovery time. For this reason, a first step in quantifying impacts in ice-hockey is establishing the validity of the impact identification using the Zephyr Impact Processor. Therefore, the purpose of this study is use video to corroborate impacts identified by Zephyr trunk-worn sensors and impact processor algorithm and determine the validity of player incurred impacts (PII) among elite national ice hockey team members. A second purpose is to characterize the nature of player incurred impacts (PII) such as falling, colliding with the boards, and the intentional act of checking where players are using their body to collide with other players to gain control of the puck. This will serve as foundational work to enable the larger-scale quantification of PII in ice-hockey.

## Methods

### Participants

From the USA Hockey National Team Development Program (NTDP) 23 male players under the age of 18 (17.5+.21 y, 1.82+0.8 m, 83.1+7.6 kg) wore Zephyr BH3 (Medtronic, MD) trunk worn sensors (TWS). The players gave written and verbal informed consent to procedures approved by Eastern Michigan University Human Subjects Committee all procedures followed the ethical guidelines for human research. In the event players were minors, parental consent and player assent was obtained.

### Instruments

Zephyr BH3 (Medtronic, MD) triaxial accelerometer trunk worn sensors (TWS) were used to measure accelerations at a sampling rate of 100 Hz and saved onboard as ACCEL files. Stored data was downloaded using Omnisense downloader application (Medtronic, MD) and raw ACCEL output files were processed using the Omnisense Impact Processor application (Medtronic, MD) on a Dell D600 laptop (Dell, TX). Impact processed output files with timestamped Impacts were then used to identify impacts of interest.

Game video was recorded by NTDP staff using video and XOS (XOS, MA) video analysis software. Game video was time synced with Impact processed files to enable visual corroboration. The observation and classification of impact events identified by the wearable sensors into the categories of impacts and their further sub-categories were conducted by one observer. The observer was trained by one of the researchers to recognize the different impact events. The researcher is a certified Level 4 USA Hockey coach with 7 years of coaching experience and a further 40+ years playing experience. Therefore, specific knowledge of on-ice events was quite credible. This background was used to train the observer to identify events such as Player incurred impacts (PII), False positives, Non-player incurred impacts, or events that were Unable to Corroborate. Observer was also trained to further subcategorized PII events as 1) Board contact/no check, as 2) Board contact/check, as 3) Open ice check, and 4) Player fall. Non-PII were also subcategorized as 5) other form of player to player event, as 6) Hard Stop, as 7) Slapshots and 8) other identifiable player events.

### Methodological procedures

All members of the team wore TWS as part of a larger project to quantify impacts and training loads over the course of the competitive season. The TWS were worn with the sensor superficial to the sternum and configured appropriately in the Omnisense Live Configuration application (Medtronic, MD) (moved from participates section. To validate impacts identified by the TWS and Zephyr Impact Processor algorithm, four games were selected that had corresponding video collected. For the four games, for practical purposes, 8 players with the highest activity levels identified by TWS were selected for impact corroboration. Impacts identified by the Zephyr impact processor software were compared to the video by timestamp to confirm the nature of the impact. The events identified by the TWS and Impact processor were categorized by frequency (N), magnitude (g), and duration (s). Events were further categorized into Zone’s (Z1-Z5) by magnitude (g), (-3.9g (Z1), 4–5.9g (Z2), 6–7.9 g (Z3), 8–9.9 g (Z4) and 10+ g (Z5)) according to the Zephyr Impact Processor criteria. Initial pilot work determined only impacts of > 6 g were relevant, so, impacts identified by WS of 6–7.9 g (Z3), 8–9.9 g (Z4) and 10+ g (Z5) were used to corroborate Player incurred impacts (PII). Accelerometer data of the top 8 active players that was categorized as a Z3, Z4, or Z5 event was time synchronized with the game video. Each event was observed visually at the corresponding time in the video footage and categorized based on magnitude.

### Data analysis

Each observed impact of the top 8 active players that was categorized by Omnisense Impact Processor as Z3, Z4, or Z5, was visually classified based on perceived mechanism of action as determined by the observer (Table 1). Events were categorized as either a Player incurred impact (PII), False positive, Non-player incurred impacts, or Unable to Corroborate (Table 1). PII events were further subcategorized as 1) Board contact/no check, as 2) Board contact/check, as 3) Open ice check, and 4) Player fall. Non-PII were also subcategorized as 5) other form of player to player event, as 6) Hard Stop, as 7) Slapshots and 8) other identifiable player events as in “Table 1”.

**Table 1:** Definitions for categorizing events observed by wearable sensors.

Event	Definition	Sub-category
Player incurred impacts	Board contact/no check	1
	Board contact/check	2
	Open ice check	3
	Player fall	4
Non-player incurred impacts	Other form of player to player event	5
	Hard Stop	6
	Slapshot	7
	Other identifiable player event	8
False positive	Event registered as a Z3, Z4 or Z5 but no discernable mechanism for the event could be observed via game footage	
Unable to Corroborate	WS observed that an event occurred but could not be corroborated e.g. Player was not on camera, not on the ice, or the event was not during game time	

Peak magnitude (g) and impact duration (s) of each impact identified from TWS were then compared by sub-category using MANOVA with Tukey post hoc (α = 0.05). Effect sizes were determined by partial eta squared. All statistical analyses were performed using SPSS 22.0 (IBM, NY).

## Results

A total of 554 events categorized as Z3, Z4, and Z5 were detected by the wearable sensor from the top 8 active players across 4 games. All events were thoroughly reviewed through video and of those 554 events 135 could not be corroborated with video footage or were not relevant for reasons such as player was not on camera, not on the ice, or the event was not during game time and were removed from further analysis. Of the remaining 419 on-ice impacts logged by the WS, 358 were confirmed true PII (85.5%), 60 were confirmed as other non-PII (14.3%) and 1 false positive (0.2%), as noted in “Table 2”.

**Table 2:** Frequency of events observed by wearable sensors by primary category.

Event	Category	Frequency (N)	% of total
Player incurred impacts (PII)	1	358	85.4%
False positive	2	1	0.2%
Non- PII	3	60	14.3%
Total		419	100.0%

Of the 358 PII, 17 (4.1%) were sub-categorized as 1) Board contact/no check, 74 (17.7%) as 2) Board contact/check, 202 (48.2%) as 3) Open ice check, 65 (15.5%) as 4) Player fall. Of the 60 Non-PII, 19 (4.5%) as 5) other form of player to player event, 16 (3.8%) as 6) Hard Stop, 19 (4.5%) as 7) Slapshots and 6 (1.4%) as 8) other identifiable player events, and only 1 false positive making the remaining 0.2% these statistics are laid out in “Table 3”.

**Table 3:** Frequency of events observed by wearable sensors by sub-category.

Event	Definition	Sub-category	Frequency (N)	% of total
Player incurred impacts	Board contact/no check	1	17	4.1%
	Board contact/check	2	74	17.7%
	Open ice check	3	202	48.2%
	Player fall	4	65	15.5%
Non-player incurred impacts	Other form of player to player event	5	19	4.5%
	Hard Stop	6	16	3.8%
	Slapshot	7	19	4.5%
	Other identifiable player event	8	6	1.4%
False positive			1	0.2%
Total			419	100%

The most prevalent impacts observed were open ice checks and board contact/check, which accounted for 202 (48.2%) and 74 (17.7%) respectively of the total WS detected events.

Of the 419 on-ice impacts logged and categorized into either Zone 3 (Z3), Zone 4 (Z4), or Zone 5 (Z5) by the WS, 160 of the 200 Z3 events were confirmed PII (80%), 103 of 110 Z4 events (93.6%) and 95 of 109 Z5 events were PII (87.2%), which can be seen in “Table 4”.

**Table 4:** Frequency of events observed by wearable sensors by zone.

Zone	Magnitude range (g)	Total events (N)	Total PII (N)	% of total
3	6-7.9	200	160	80.0%
4	8-9.9	110	103	93.6%
5	10+	109	95	87.2%
Total		419	358	

At higher magnitudes (g) of impacts WS had a stronger level of agreement in identifying true PII 160 (80%) of 6–7.9 g (Z3), 103 (93.6%) 8–9.9 g (Z4) and 95 (87.2%) 10+ g (Z5).

**Fig 1:**
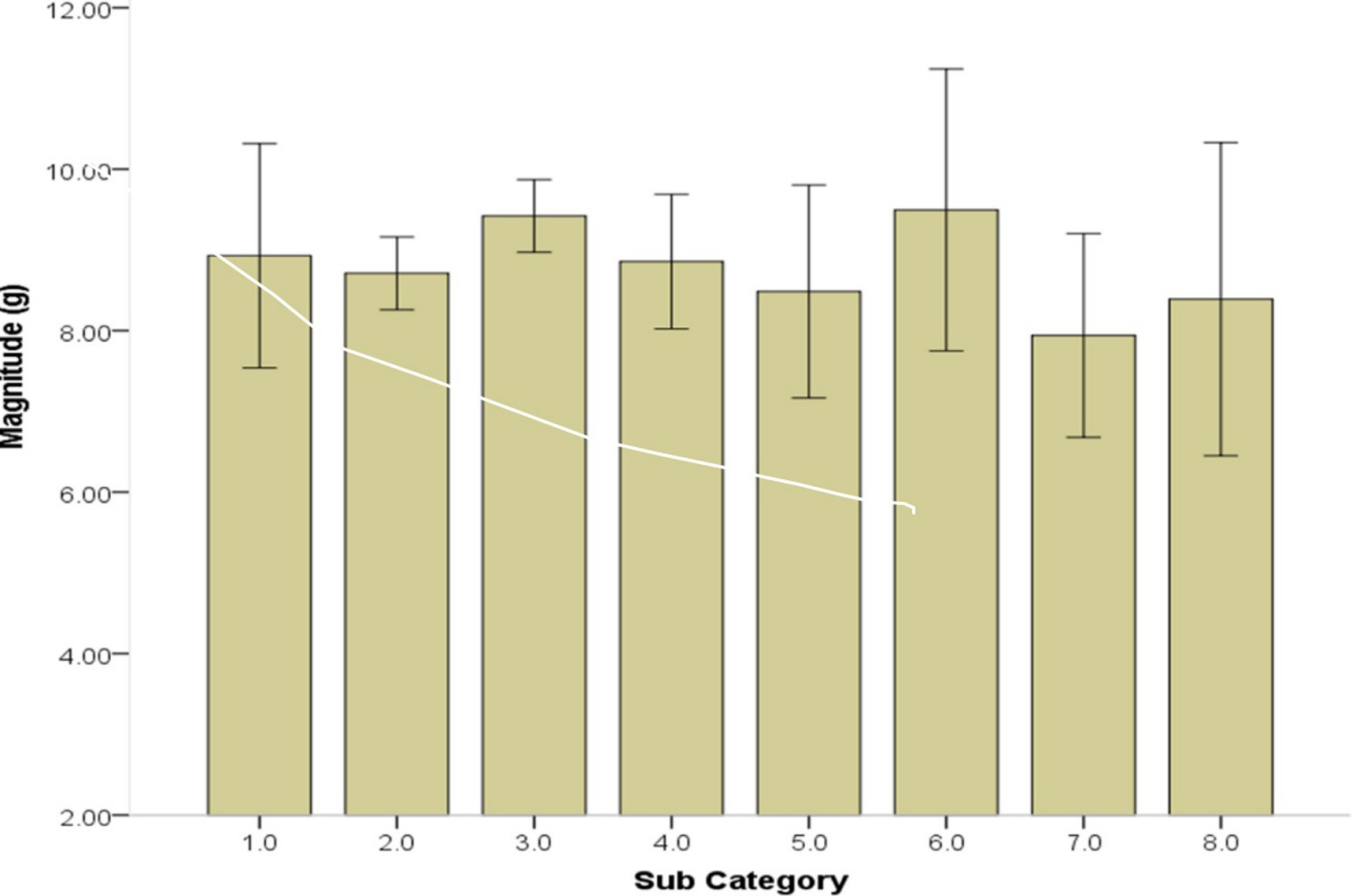
Mean magnitudes of events observed by wearable sensors. Of the 358 player incurred impacts (PII) and 60 Non-PII logged by the wearable sensors (WS) of the top 8 active players across 4 games were compared by sub-category in terms of the magnitude (g) of the recorded impact. Sub-category (1) Board contact/no check, (2) Board contact/check, (3) Open ice check, (4) Player fall, (5) other form of player to player event, (6) Hard Stop, (7) Slapshots and (8) other identifiable player events.

When comparing the sub-categories of the 358 PII and 60 Non-PII events logged by the WS the magnitude (g) of impacts was not significantly different by sub-category as seen in “Fig 1”. Although not significant, there was a small effect for duration (partial eta squared = 0.02).

**Fig 2:**
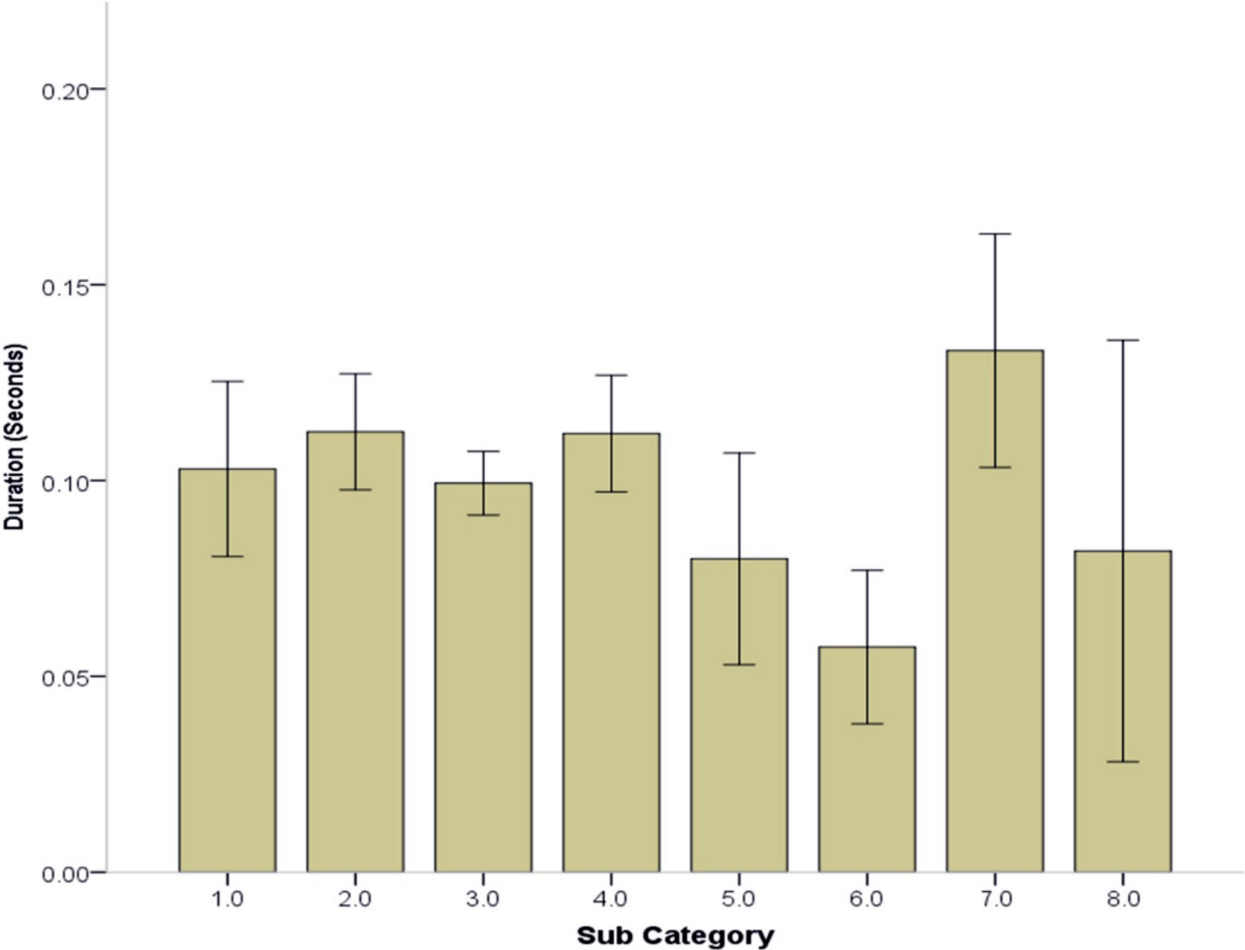
Mean durations of events observed by wearable sensors. Of the 358 player incurred impacts (PII) and 60 Non-PII logged by the wearable sensors (WS) of the top 8 active players across 4 games were compared by sub-category in terms of the duration (seconds) of the recorded impact. Sub-category (1) Board contact/no check, (2) Board contact/check, (3) Open ice check, (4) Player fall, (5) other form of player to player event, (6) Hard Stop, (7) Slapshots and (8) other identifiable player events.

When comparing the sub-categories of the 358 PII and 60 Non-PII events logged by the WS the duration of category 6 (Hard stop; .058 s) was lower than sub-categories 2, 4 and 7 (.112, .112, .133 s, respectively, p < .05) as seen in “Fig 2”. This significant result exhibited a moderate effect ((partial eta squared = 0.052)

With regard to reliability of the data, when analyzed across all four games, there were no significant differences for magnitude (p = .781) or duration (p = .437) of observed impacts. Effects were trivial for both (partial eta squared = 0.003 and .007, respectively). The trivial effect size indicates that the lack of significance was not due to lack of sufficient sample size, but related to the similarity of the data across games.

## Discussion

The purpose of this study was to use video to corroborate impacts identified by trunk-worn wearable sensors (WS) and determine validity of player incurred impacts (PII) among elite national ice hockey team members. The findings indicate that wearable sensors have a greater validity of detecting Ice-hockey player incurred impacts at higher magnitudes events. Of the total 419 on-ice impacts logged by the WS, 358 were confirmed true PII (85.5%), when broken down by zone 160 of the 200 Z3 events were confirmed PII (80%) but validity of PII detection increases by over 10% at the higher magnitude Zones with 103 of 110 Z4 events (93.6%) and 95 of 109 Z5 events were PII (87.2%).

With the intention to automatically detect player incurred impacts at high magnitude zones (Z3, Z4, and Z5) the WS detected 419 on-ice impacts, after reviewing the events with video corroboration 358 were confirmed true PII (85.5%), 60 were confirmed as other non-PII (14.3%) and 1 false positive (0.2%). From another perspective, 418 (99.8%) of the 419 events were unique identifiable events that could be sub-categorized as impacts of some nature. Of the 358 PII, 17 (4.1%) were categorized as 1) Board contact/no check, 74 (17.7%) as 2) Board contact/check, 202 (48.2%) as 3) Open ice check, 65 (15.5%) as 4) Player fall Of the 60 Non-PII, 19 (4.5%) as 5) other form of player to player event, 16 (3.8%) as 6) Hard Stop, 19 (4.5%) as 7) Slapshots and 6 (1.4%) as 8) other identifiable player events. Therefore, trunk worn WS with the use of the impact identification algorithm were quite effective at identifying on-ice impacts in an automated fashion in an ecologically valid setting.

Of particular interest was the occurrence of actual checking events within the pool of PII identified by the WS. To this point, the most prevalent impacts observed were those where there was a contest for control of the puck; open ice checks and board contact/check, which accounted for 202 (48.2%) and 74 (17.7%) respectively of the total WS detected events. Therefore, the act of checking accounted for 276 (65.9%) of the total WS detected events. Interestingly a similar pattern was observed by Gastin et al. who examined the validity of WS to detect tackles in Australian rules football. These authors observed that 1125 (75%) of the total 1510 (100%) true impacts were instances of player contact in which the control of the ball was being contested [19]. Since it appears as though impacts will likely occur as a function of competition for the puck in ice-hockey, a question for future studies will pertain to the incidence of contacts in checking vs non-checking levels. It is plausible that PII will still occur as a function of competition for the puck, but the frequency and magnitude of such impacts may be different in checking vs non-checking levels.

In the current study it was also noted that at higher magnitudes (g) of impacts WS had a stronger level of agreement in identifying true PII 160 (80%) of 6–7.9 g (Z3), 103 (93.6%) 8–9.9 g (Z4) and 95 (87.2%) 10+ g (Z5). A similar trend was also reported by Cortes et al in their study of the validation of wearable sensors to detect impacts in the sport of lacrosse. They observed that a greater percentage of impacts were verified as true impacts at higher magnitudes for both boys and girls. It was reported that 57% and 25% verified impacts at the lowest percentile magnitude occurred vs 74% and 50% verified impacts at the highest percentile magnitude for boys and girls, respectively [20]. Other investigators also reported a similar trend when Gastin et al studied wearable sensor validity for the detection of tackles in Australian rules football. In this study, the magnitude of impacts was represented in terms of player load and impacts were categorized as low, medium or high intensity. Despite these technical differences, the authors observed that WS correctly detected only 58 (50%) of a total 115 low intensity impacts (tackles) but were more successful at medium (198 (92%) of 216) and high intensity (19 (90%) of 21)[19]. So, it appears that regardless of the nature of the activity, (running or skating) or method (g’s vs player load), accelerometer based WS are more successful at identifying impacts at higher magnitudes.

The present study indicated that the magnitude of impacts was not significantly different between categories, however, statistically, the duration (s) of category 6 (Hard stop; .058 s) was shorter than categories 2, 4 and 7 (.112, .112, .133 s, respectively, p < .05). The observation of shorter duration events for hard stops vs. impacts is likely due to the more complex nature of collisions between multiple individuals and/or the boards as opposed to the simple act of stopping. Regardless, this distinction can be exploited to improve the automated identification of PII in ice-hockey. From a practical standpoint, this will allow the determination of impacts in ice-hockey using wearable sensors on a larger scale, which in turn will provide data to inform various stakeholders and policy makers with regard to the number and magnitude of impacts incurred by players at various levels of the sport.

Limitations present in this study include the observation and analysis of only one of the two competing teams via trunk worn sensors. This was unavoidable as only one team was equipped with sensors. This may be addressed in future studies with more extensive distribution of TWS. Another limiting factor was the relatively small subject sample size. These sensor systems are fairly expensive and therefore difficult to use on a large scale due to financial considerations. With the rapid progress of technology and proliferation of wearable sensors though, larger scale projects should be feasible in the near future. Research in the future may benefit by the incorporation of machine learning, and sensor fusion, to improve the accuracy of automated identification of player incurred impacts using wearable sensors. With automated identification, PII occurring in ice-hockey can be quantified and qualified on a large scale. This large-scale characterization of PII may be used to better inform the debate around checking and/or address specific issues of player safety in ice-hockey. As well, with larger scale, automated studies, it would be feasible to perform more sophisticated analyses such as comparing the difference in impacts between positions or between practices and games.

## Conclusion

These data show that using some limited criteria (e.g. impact magnitude and duration), PII can be identified with relatively high accuracy in ice hockey using trunk-worn wearable sensors. Of particular interest was the finding that wearable sensors detected impacts of higher magnitudes with a greater degree of accuracy. Approximately 65% of detected events were related to checking, as either open ice checks and board contact/check, which accounted for 202 (48.2%) and 74 (17.7%) respectively. It was noted that while the magnitude of impacts was not different by category, but the duration of category 6 (Hard stop; .058 s) was lower than categories 2, 4 and 7 (.112, .112, .133 s, respectively, p < .05). From a practical standpoint, by validating the use of wearable sensor technology for the quantification of impacts in the sport of Ice Hockey, it will be possible to determine the impacts incurred by players on a much larger scale and provide quantitative data in this regards to athletes, coaches and governing bodies as has been accomplished in other sports such as Australian rules Football and lacrosse.

## Supporting information

S1 Dataset Wearable sensor data. (XLSX)

S1 Video Board contact no check. (MP4)

S2 Video Board contact check. (MP4)

S3 Video Open ice check. (MP4)

S4 Video Player fall. (MP4)

S5 Video Other player to player interaction. (MP4)

S6 Video Hard stop. (MP4)

S7 Video Slapshot. (MP4)
